# Extracellular vesicles: novel communicators in lung diseases

**DOI:** 10.1186/s12931-020-01423-y

**Published:** 2020-07-08

**Authors:** Aradhana Mohan, Stuti Agarwal, Matthias Clauss, Nicholas S. Britt, Navneet K. Dhillon

**Affiliations:** 1 Division of Pulmonary and Critical Care Medicine, Department of Internal Medicine, University of Kansas Medical Center, Mail Stop 3007, 3901 Rainbow Blvd, Kansas City, KS 66160 USA; 2 Division of Pulmonary, Critical Care, Sleep & Occupational Medicine, Indiana University School of Medicine, Indianapolis, Indiana USA; 3 Department of Pharmacy Practice, University of Kansas School of Pharmacy, Lawrence, Kansas USA; 4 Division of Infectious Diseases, Department of Internal Medicine, University of Kansas Medical Center, Kansas City, Kansas USA; 5 Department of Molecular & Integrative Physiology, University of Kansas Medical Center, Kansas City, Kansas USA

## Abstract

The lung is the organ with the highest vascular density in the human body. It is therefore perceivable that the endothelium of the lung contributes significantly to the circulation of extracellular vesicles (EVs), which include exosomes, microvesicles, and apoptotic bodies. In addition to the endothelium, EVs may arise from alveolar macrophages, fibroblasts and epithelial cells. Because EVs harbor cargo molecules, such as miRNA, mRNA, and proteins, these intercellular communicators provide important insight into the health and disease condition of donor cells and may serve as useful biomarkers of lung disease processes. This comprehensive review focuses on what is currently known about the role of EVs as markers and mediators of lung pathologies including COPD, pulmonary hypertension, asthma, lung cancer and ALI/ARDS. We also explore the role EVs can potentially serve as therapeutics for these lung diseases when released from healthy progenitor cells, such as mesenchymal stem cells.

## Extracellular vesicles

Cell-to-cell communication is essential for nearly all physiologic and metabolic processes. This intercellular conveyance is achieved through receptor ligands, signaling molecules, hormones, and extracellular vesicles (EVs). Historically, secretion of the cell in the form of EVs was considered as unimportant waste material, cellular “garbage bags,” or dust particles [1–5]. However, in recent years, this so-called “waste” is now known to be of profound importance in various biological systems, creating a boon in their exploration across the scientific community.

**Fig. 1:**
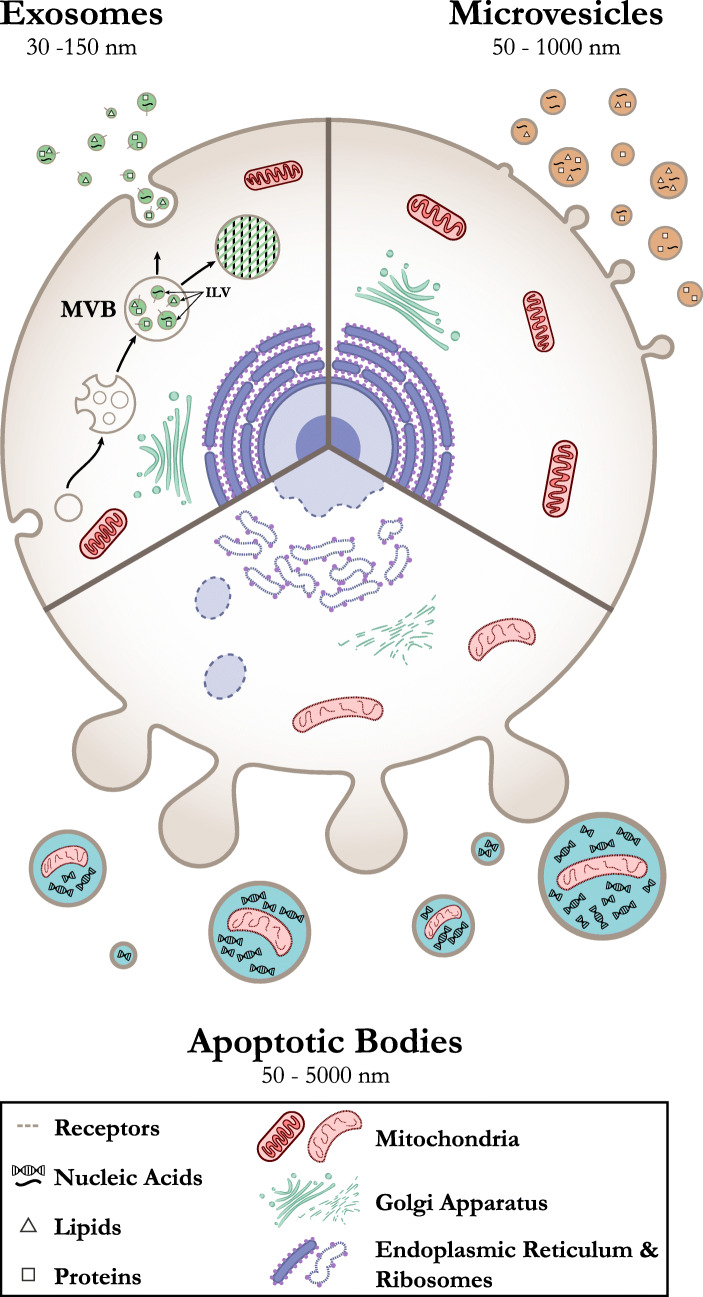
Biogenesis of various forms of extracellular vesicles from a eukaryotic cell. Exosomes are generated through multivesicular bodies (MVB) and intraluminal vesicles (ILV) formation whereas microvesicles/microparticles and apoptotic bodies are vesicles generated through blebbing of plasma membrane

Lipid bilayer membrane-enclosed vesicles are secreted by both prokaryotic and eukaryotic cells [4–9]. Although, the term “extracellular vesicle” is sometimes used in reference to exosomes, it is actually a very broad term that encompasses all different types of vesicles secreted outside the cells [10]. Regardless, the function of all these vesicles appears to be all the same: communication between the cells within an organism or between species [11, 12]. In addition, it is not necessary that all vesicles secreted from cells are functional or have any role in some kind of biological process. Sometimes, they just act as “dustcart” to remove the waste from cells [13]. Of note, there have been discrepancies in the classification of these vesicles in the literature. Some studies divide EVs into two major categories: I) exosomes, defined as vesicles released by exocytosis of the multivesicular bodies; and II) ectosomes, defined as the vesicles which are assembled and released by the plasma membrane [14]. However, most recent studies categorize EVs as either exosome, microvesicles, microparticles, or apoptotic bodies based on vesicle size and how they are formed [15–21] (Fig. 1).

### Exosomes

Exosomes are small EVs with sizes ranging between 30 and 150 nm in diameter that originate from the internal vesicles of multivesicular bodies (MVB) of nearly all cell types. Exosomes originating from different cell types have different composition; however, there are certain characteristics, which are common to all exosomes regardless of their source. They usually sediment between ~ 70,000–200,000 x g and their molecular cargo consists of proteins, lipids and nucleic acid molecules [22–24]. There are two basic mechanisms reported for the formation of MVBs and intraluminal vesicles (ILVs) leading to exosome generation: I) ESCRT-dependent [24] [23]; and ii) ESCRT-independent [25, 26]. Details of these mechanisms have been explained previously in multiple publications [26–36].

A typical exosome is surrounded by a phospholipid membrane that contains lipids characteristic of their cellular origin [37, 38] with high levels of cholesterol, sphingomyelin, and ceramide and detergent-resistant membrane domains (lipid rafts) [39, 40]. Also present are proteins associated with lipid rafts, such as glycosylphosphatidylinositol-anchored proteins and flotillin [39, 41]. Some lipids are present in greater amounts in exosomes compared to their parent cells, thus improving the rigidity of the exosomal membrane [42, 43]. Components of the ESCRT complex, such as Alix and tumor susceptibility gene 101 (TSG101), involved in MVB biogenesis [44] [45] [46], are distinguishing proteins present on exosomes [47, 48]. Another distinguishing feature of exosomes is the presence of tetraspanins, including CD9, CD63, CD81 and CD82 [49]. Other proteins present in exosomes includes cytosolic proteins, such as Rabs, which are involved in promoting exosome docking and membrane fusion events [50, 51], as well as annexins, which are proteins believed to regulate membrane cytoskeleton dynamics and membrane fusion events [50]. Myriad studies have shown the presence of nucleic acid cargo [49, 52–54] within exosomes that are functionally active when released in the recipient cells. This nucleic acid cargo may include a variety of non-coding RNAs including microRNA and long non-coding RNA (lncRNA), tRNA fragments, small-interfering RNAs, structural RNAs, small RNA transcripts and RNA-protein complexes. Other than different RNA species, exosomes also contain DNA which could represent the entire genome as well as the genomic mutations, making them excellent biomarkers [55–58]. In addition to chromosomal DNA, mitochondrial DNA has also been reported [59, 60].

### Microvesicles

Microvesicles (MVs), or microparticles (MPs) (50–1000 nm), are secreted by direct outward budding of the plasma membrane of living cells with release of membrane microvilli [61, 62]. These vesicles are generally larger in size up to ~ 1000 nm [18], but smaller vesicles (~ 50 nm) also bud from the plasma membrane [63]. Microvesicles have also been reported in various shapes. Typical markers used for detecting MVs are integrins, selectins, and CD40 [62]. However, various other markers may be used dependent on the cell type from which they are secreted. Studies also suggest that the vesicles which sediment at ~ 10,000–20,000 x g represent microvesicles [16, 64, 65]. Since microvesicles are shed by the budding of the plasma membrane, their composition is same as that of plasma membrane, except that the lipid composition is uniformly distributed across the bilayer membrane of the microvesicles, in contrast to the asymmetrical distribution present on the two leaflets of the plasma membrane [66–68]. Although the shedding of MVs from cells takes place at resting state, some cells release MVs based upon the stimulant they receive. Purinergic receptors, P2Y receptors, phorbol esters, and calcium have been reported to be involved in the robust release of MVs [69–73].

### Apoptotic bodies

Apoptotic bodies (also referred to as “apoptotic blebs” or “apoptotic vesicles”) represent type of EVs released by the outward budding, blebbing, or fragmentation of the plasma membrane during the apoptosis of cells. These vesicles are generally larger in size, ranging from 50 nm to 3 μm [62], with some studies suggesting sizes ranging from 1 to 5 μm [24, 65, 74–76]. The content of apoptotic bodies released from plasma membranes differs depending on cellular origin, but has been shown to contain DNA fragments and histones [65]. Apoptotic bodies originating from the endoplasmic reticulum are devoid of DNA and histones, but contain immature glycoepitopes [65, 77, 78]. When these vesicles are taken up by antigen-presenting or neighboring cells, it may lead to anti-inflammatory or tolerogenic response [1, 79, 80].

With the exponential growth of EV research in recent years, this review attempts to comprehensively update and highlight the importance of EVs with regard to various pulmonary complications and disease states.

## Role of EVs in the pathogenesis of lung diseases

### Chronic obstructive pulmonary disease (COPD)

Chronic obstructive pulmonary disease is characterized by severe airway inflammation and subsequent damage of the lung parenchyma. This hyper inflammatory response leads to destruction of the alveolar wall and rarefaction of the alveolar sacs, causing difficulty in breathing and reduced pulmonary function, measured by total lung capacity and forced expiratory volume (FEV) [81]. The inflammatory response is most commonly incited by inhalation of toxic particles, chemical and radiological irritants, or cigarette smoke. Infections, which activate the toll like receptors (TLRs) present on resident lung cells, including alveolar macrophages, dendritic cells, alveolar epithelial cells and endothelial cells, may also contribute by stimulating the release of cytokines and chemokines [82]. Aside from the inflammatory processes of COPD, cells undergo senescence and aging (contributing to the senescence associated secretory phenotype [SASP]) [83] and release EVs that further contribute in the pathogenesis and progression of the disease. Additionally, high number of microparticles derived from platelets, red blood cells and leukocytes are also associated with chronic COPD [84, 85] (Table 1**)**.

**Table 1:** Potential extracellular vesicle markers in various lung complications

Disease subtype	EV Source	Biomarkers	Ref.
**COPD**			
	Plasma	**↑**E-selectin-, VE-cadherin, PECAM positive MPs	[84–89]
	Plasma	Ceramide levels in EMPs	[90]
	Plasma	**↑**Platelet derived LMPs and EMPs	[85]
**Pulmonary Hypertension**			
Group I PAH (Idiopathic /Heritable/ connective tissue associated PAH)	Plasma	**↑**CD39 expression and ATPase/ADPase activity	[91]
	Plasma	Translationally controlled tumor protein (TCTP) in endothelial derived EVs	[92]
	Plasma	**↑**Endothelium derived exosomes	[93]
	Plasma	**↑**EMPs (CD31^+^ & CD41^-^), Small Platelet derived MPs(CD31^+^/CD41^+^)	[94]
	Plasma	**↑**EMPs and LMPs	[95]
	Plasma	**↑**CD62e + EMPs associated with adverse outcome	[96]
	Urine	EMPs	[97]
Group III PH	Plasma	**↑**Tissue factor and endoglin in EMPs	[98]
Group IV PH	Plasma	**↑**Tissue factor and endoglin in EMPs	[98]
	Plasma	**↑**PMPs, LMPs and EMPs	[99]
**Asthma**			
	Blood	**↑**Eosinophil derived exoxomes	[100, 101]
	BALF	**↑** CD63 and CD81^+^ exosomes carrying leukotrienes biosynthesis enzymes	[102]
	BALF	**↑**Mitochondria/mitochondrial DNA in exosomes	[103]
	BALF	↓Phosphatidylglycerol, ceramide-phosphates, and ceramides	[104]
		**↑**Sphingomyelin in exosomes	
**Lung cancer**			
Late stage human lung cancer	Serum	**↑**Vimentin in exosomes	[105]
Non-small Cell Lung Cancer		**↑**lncRNA MALAT-1 in exosomes	[106]
**ALI/ARDS/ Pulmonary Sepsis**			
ARDS	BALF /plasma	**↑**LMPs associated with better survival	[107]
ARDS	Plasma	**↑**Gasdermin D in MPs	[108]
ALI	Serum	Apoptosis-associated speck-like protein containing a caspase-recruiting domain (ASC) in EVs	[109]
ARDS	Pulmonary edema fluid	Tissue Factor in MPs	[110]
Sepsis / Community-acquired pneumonia -sepsis	Plasma	**↑**Alpha-2-macroglobuin in MVs/MPs correlates with survival	[111, 112]

Endothelial derived MPs (EMPs), Platelet-derived MP (PMPs), Leukocyte derived MPs (LMPs)

Multiple studies have described mononuclear/macrophage-derived EVs rich in inflammatory effector molecules like cytokines, chemokines, adhesion molecules and proteases to cause alveolar wall destruction and emphysema, the hallmark pathological features of COPD [113–115]. Cigarette smoke exposure causes increased release of tissue factor (TF)-positive microvesicles with high pro-coagulant activity from human macrophages [115]. These EVs also carry MMP14 that is responsible for promoting lung emphysema via its collagen degradation and gelatinolytic properties [116]. Another study described increased release of macrophage-derived EVs containing IL-8, MCP-1 and ICAM-1 pro-inflammatory molecules on the activation of monocytes by cigarette smoke exposure [114].

A recent study suggested that the COPD pathogenesis is promoted via neutrophil elastase coated exosomes released by activated but not quiescent polymorphonuclear leukocyte neutrophils. The neutrophil elastase linked to exosomes destroys the extracellular matrix proteins of the alveoli leading to the development of COPD like characteristics [117].

Chronic obstructive pulmonary disease is also characterized by endothelial cell damage due to increased apoptosis. Microparticles containing endothelial markers, such as CD31, CD62E (E-selectin), CD143, and CD105, are differentially released from these apoptotic endothelial cells based on the pathophysiological stage of the disease [84, 116] (Table 1**)**. These microparticles are believed to promote progression of COPD by causing apoptosis of neighboring healthy endothelial cells upon delivery of inflammatory cargo [116]. Chronic obstructive pulmonary disease is also one of the major secondary complications that arise in human immunodeficiency virus (HIV)-infected patients [118]. Interestingly, it has recently been reported that EVs isolated from bronchoalveolar lavage (BAL) fluid of HIV-positive patients carry HIV-Nef protein that induces endothelial cell apoptosis, which may be responsible in promoting emphysema and pulmonary vascular changes observed during COPD [118]. As such, further research into the role of EVs as mediators of the chronic pulmonary complications of HIV infection appears to be well warranted.

**Fig. 2:**
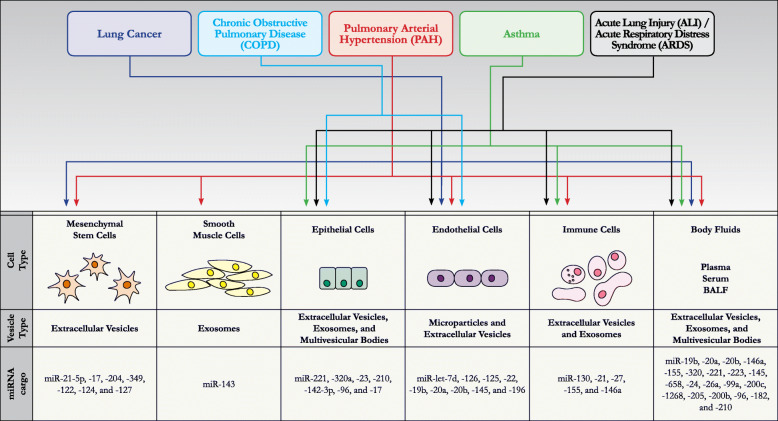
Illustration showing release of various types of extracellular vesicles and their miRNA content released from different cell types/body fluids in various lung complications

Cigarette smoke exposure also leads to increased release of microparticles from mouse and human microvascular and pulmonary arterial endothelial cells [90]. These microparticles are enriched in ceramides and phosphatidyl serine due to high sphingomyelinase activity in endothelial cells, crucial for stress induced apoptosis [90]. These endothelial-derived microparticles (EMPs) circulate in the blood and can act as a biomarker to assess the severity of the disease condition [90, 119] (Table 1**)**. Serban et al. reported a greater number of EMPs in plasma of COPD patients who were cigarette smokers as compared to healthy non-smokers [90]. These cigarette smoke-associated EMPs had high levels of miRNAs such as let-7d, miR− 126, − 125-5p, and − 22 that have been known to promote angiogenesis, airway inflammation, cancer progression, cell cycle arrest, and apoptosis [90] (Fig. 2). Direct exposure of cigarette smoke on primary human microvascular endothelial cells led to release of microparticles that were reported to impair macrophage efferocytosis (clearance of apoptotic cells), thus exacerbating the inflammation and endothelial injury [90]. In another study carried out on COPD patients, the number of CD31^+^ EMPs suggestive of endothelial apoptosis was found to be elevated in plasma of patients with mild COPD that also exhibited emphysema [86]. Furthermore, in patients with severe COPD, CD62^+^ EMPs were found to be increased due to endothelial activation during severe form of disease [86]. A subsequent study elaborated on the exaggerated release of CD31^+^ EMPs in the plasma of cigarette smokers with COPD or no COPD, in comparison to non-smokers [87]. As COPD patients undergo excessive levels of lung endothelial damage and persistent endothelial stress with no improvement in lung function, cessation of smoking did not change the plasma CD31^+^ EMPs levels in smokers with COPD patients, whereas reduction in the release of EMPs was observed in smokers without COPD [87]. In another prospective study of COPD patients in Japan, the number of VE-cadherin (CD144^+^) EMPs, CD31^+^ EMPs, and E-selectin (CD62^+^) EMPs were evaluated in blood samples and correlated with FEV over 1 s (FEV_1_) changes in the lung [88]. It was shown that an increase in circulating VE-cadherin EMPs positively correlated with FEV decline in these patients, suggesting endothelial injury to be a prominent factor in the development of COPD [88]. In another study, increased levels of CD31^+^, CD62^+^ and CD144^+^ EMPs were found in the blood of COPD patients as compared to healthy controls [89]. Specifically, circulating E-selectin and VE-cadherin EMPs were found to be associated with increased exacerbation susceptibility and decline in FEV and total lung capacity in severe COPD patients [89] (Table 1**)**. Finally, in a study by Liu et al., rats exposed to cigarette smoke for 2–6 months showed elevated levels of circulating CD31^+^ and CD62E+ EMPs in the blood plasma. The elevated level of EMPs was found to be proportional to lung destruction and corresponded to a decrease in pulmonary function parameters such as forced vital capacity and total lung volume [119].

In COPD induced by cigarette smoke exposure, alveolar macrophages and alveolar epithelial cells are directly exposed to the toxic components of cigarette smoke [120]. The role of lung epithelial cell-derived EVs in the pathogenesis of COPD has also been well reported [120]. Cigarette smoke exposure leads to exaggerated release of exosomes from damaged epithelial cells and promotes emphysema [120]. It has been shown that human bronchial epithelial cells (BEAS-2B) exposed to cigarette smoke demonstrate increased Rab27A-dependent release of EVs [121]. Cigarette smoke exposure in bronchial epithelial cells also stimulates release of full-length CYR61/CTGF/NOV family1 (flCCN1)-enriched EVs which mediate IL-8 induced inflammation, but also helps maintain lung homeostasis by increasing the levels of vascular endothelial growth factor (VEGF) [121]. However, prolonged cigarette smoke exposure causes cleavage of flCCN1 to form CCN1 in the extracellular matrix, thereby promoting the release of MMPs and reduced VEGF, causing epithelial cell damage and development of emphysema [121]. A recent study by Fujita et al. demonstrated that cigarette smoke extract induced human bronchial epithelial cell-derived EVs promote myofibroblast differentiation of the lung fibroblasts, leading to the development of fibrosis [122]. The bronchial epithelial cell-derived EVs carry miR-210 that regulates autophagy by directly targeting Atg7 [122]. Reduction in autophagy by miR-210 mediated Atg7 inhibition leads to differentiation of lung fibroblasts into myofibroblasts [122]. Furthermore, lung fibroblasts isolated from COPD patients demonstrate decreased autophagy and fibroblast differentiation [122]. Epithelial-derived exosomes following cigarette smoke exposure are also known to carry pro-inflammatory cytokines and Wnt-5a that are delivered to neighboring and far away cells via circulation [120].

### Pulmonary hypertension (PH)

Pulmonary hypertension is a chronic progressive disease that leads to right ventricular failure and ultimately death. The prime pathological feature of the pulmonary hypertension is vascular remodeling in lungs leading to the loss of endothelial integrity and proliferation of vascular smooth cells [123, 124]. The resulting increase in the thickness and stiffness of blood vessels leads to chronic increase in mean pulmonary artery and right ventricular systolic pressures, ultimately resulting in right ventricular failure [123, 124]. Lately, a number of studies have demonstrated the pivotal role EVs serve in the progression and prevention of PH, as well as useful biomarkers of the disease.

An early study published by Bakouboula et al. in 2008 showed an increase in endothelium-derived CD105 microparticles in pulmonary arterial blood from patients with pulmonary arterial hypertension (PAH) or Group 1 PH [98]. Another study shortly thereafter reported an increase in the levels of microparticles positive for endothelial PECAM and VE-cadherin (but not E-selectin) in the plasma samples from PAH patients compared to controls [95]. This increase in EMPs was found to be correlated with the increase in mean pulmonary arterial pressure, pulmonary vascular resistance, and mean right arterial pressure [95]. The same group later demonstrated higher number of circulating CD62e ^+^ EMPs in the plasma of PH patients [96] (Table 1**)**.

Later Diehl et al. observed that the platelet activation and inflammation during thromboembolic PH results in increased levels of platelet and leukocyte-derived microparticles in blood [99]. Furthermore, increased levels of circulating EMPs were observed in these patients due to enhanced endothelial apoptosis indicative of thromboembolic complications and PH progression [99]. Additionally, the circulating platelet- and endothelial-derived microparticles from plasma of idiopathic PAH patients (IPAH) showed the presence and increased expression of CD39 and ATPase/ADPase activity compared to those from healthy controls [91]. CD39 (ENTPD1), an ectonucleotidase responsible for extracellular dephosphorylation of ATP to ADP and AMP, leads to activation of purinergic cell signaling pathways involved in PAH pathogenesis [91]. Further research found increased levels of small platelet-derived microparticles (PMP) and EMPs in plasma from patients with either IPAH, heritable PAH or PAH associated with connective tissue diseases, suggesting a common phenomenon of inflammation and vascular dysfunction occurs in the progression of all forms of PAH [94]. Moreover, an increased level of endothelial, CD31^+^ derived exosomes in the plasma of patients with idiopathic PAH was also found [93] (Table 1**)**. The study also noted an important observation that the exosomes released by human pulmonary artery endothelial cells in response to inflammation and hypoxia, when administered to human pulmonary arterial smooth muscle cells (HPASMCs), induce proliferation in these recipient cells [93]. Among the PAH patients with other complications such as transfusion-dependent β-thalassemia and hemoglobin E thalassemia, the presence of increased phosphatidylserine (PS)-containing red blood cell- and platelet-derived EVs were found [125].

Among the various pre-clinical animal model studies, the pathological role of EV was beautifully described in the mice model of monocrotaline-induced PAH (MCT-PAH) by Aliotta et al. They reported that the injection of lung- and plasma-derived small-sized EVs (30–400 μm) isolated from the diseased mice is able to develop PAH in the healthy mice [126]. They reported increased levels of miRs-19b,-20a,-20b, and − 145 known to be targeting BMPR signaling, apoptosis and cell proliferation in the exosomes from MCT mice and IPAH patients [126] (Fig. 2). In the same mice model, it was demonstrated that EVs from bone marrow, lung, and plasma reduced apoptosis of pulmonary vascular endothelial cells (PVECs) [127]. Hypoxia-induced miR-107, responsible for decreasing pro-apoptotic signal, was increased in these cells after treatment with the EVs from the PAH mice model [128]. Not only this, EVs released by PVECs from MCT-PAH mice can convert healthy bone marrow derived endothelial progenitor cells into a pathological progenitor phenotype [129]. When injected into the lungs of healthy mice, these pathological progenitors cause pulmonary vascular remodeling [129].

Circulating platelet and erythrocyte-derived MPs from hypoxic rats are known to decrease nitric oxide production and increase xanthine oxidase and mitochondrial reactive oxygen species (ROS) in pulmonary arterial endothelial cells, further contributing to their dysfunction [130]. This is executed via MP-driven reduction in endothelial nitric oxide synthase (eNOS) activity highly specific to pulmonary ECs [130]. Moreover, endoglin positive MPs from the blood of the Sugen-hypoxia rat model of severe PAH induced the expression of inflammatory adhesion molecule-1 (ICAM-1) only in pulmonary artery endothelial cells, but not pulmonary microvascular endothelial cells (PMVECs) [131]. This suggests that MPs from severe PAH contribute to pulmonary vascular lesion formation by specifically targeting pulmonary arteries [131].

Both protein and nucleic acid EV cargo have been suggested to play roles in the pathogenesis of PAH. Translationally controlled tumor protein (TCTP), known to be associated with heritable PAH, was found to be highly expressed in exosomes released from blood outgrowth endothelial cells (BOECs) with BMPR-2 mutation, [92]. In addition, TCTP in BOEC-derived exosomes is able to get transferred to PASMCs leading to SMC proliferation [92].

Furthermore, miR-143-3p induces migration and proliferation of pulmonary arterial endothelial cells (PAECs) upon its delivery via PASMC-derived exosomes, therefore contributing to PAH [132]. In a recent study from our lab, we have shown that extracellular derived vesicles obtained from HIV-infected monocyte-derived macrophages (MDMs) exposed to drugs of abuse like cocaine are able to induce human pulmonary arterial smooth muscle cell (HPASMC) proliferation [133]. We reported that these EVs carry miR-130 that target PTEN, thereby activating PI3/AKT signaling in HPASMCs leading to hyperproliferation [133]. Furthermore, disrupted signaling between pericytes and neighboring pulmonary microvascular endothelial cells (PMVECs) suggested in PAH [134] was recently reported to be associated with the reduced levels of Wnt5A ligand in exosomes secreted by PMVECs in PAH patients [135]. Overall, the EVs have the ability to induce the disease phenotype in healthy cells through regulation of various pathological pathways on transfer of its cargo.

Another potential of EVs as biomarker was supported by a study demonstrating reduced levels of miR-150 in whole plasma and plasma-derived MVs of PAH patients, which were associated with reduced survival of these patients [136]. This study highlighted the utility of plasma and plasma-derived MVs miR-150 levels as potentially clinically useful biomarkers for PAH prognosis [136]. Rose et al. showed that endothelial cell-derived MPs can act as biomarkers for right ventricular function in PAH patients, wherein the number of these particles in urine was found to be increased in PAH patients compared to healthy controls [97] (Table 1**)**. A correlation between these urine MP biomarkers and tricuspid annular plane systolic excursion (TAPSE) was observed in PAH patients [97].

### Asthma

Asthma is an inflammatory disorder characterized by pulmonary obstruction and difficulty in breathing due to airway hyper responsiveness (AHR) and airway remodeling. AHR that precedes the inflammatory response [137] is characterized by abnormal narrowing of airways due to smooth muscle hypertrophy, increased angiogenesis and fibrosis [138–142]. Broadly on the basis of inflammatory trigger, asthma could be divided into two types- extrinsic/allergic and intrinsic asthma. In extrinsic asthma, the inflammatory response is usually triggered by allergens or air pollutants like smoke, dust, or pollens [143]. However, intrinsic asthma is asthma caused by anything else other than allergens and that could be due to an internal infection, stress, exercise or weather conditions [144, 145]. Also, intrinsic asthma could be more severe compared to extrinsic asthma and may not respond to the conventional therapies [146]. However, the inflammatory response is almost same in both types of asthma. Infiltration of eosinophils, mast cells, and Th2 cells in the airways and release of pro-inflammatory effector molecules like IL-4, IL-5 and IL-13 lead to prominent structural changes and remodeling of the airways [143, 147]. The lung epithelial cells, such as bronchial epithelial cells, alveolar epithelial cells, and lung fibroblasts, maintain the structure of the airway and lung homeostasis. These cells also respond to the allergens and generate an inflammatory response by releasing cytokines that further contribute to the progression of asthma [148, 149]. This response, including secretion of IL-6, also triggers the proliferation of smooth muscle cells in the airways that causes pulmonary obstruction in asthma [150]. One of the major reasons for severe asthma are Th17 cell types which differentiate from naive CD4^+^ cells on stimulation with IL-1β, IL-23, TGF-β, and IL-6 [151–154]. Th17 cells release IL-17 and other cytokines and chemokines reported to be present in the BALF fluid of severe and moderate asthma patients [155, 156]. Binding of IL-17A on airway epithelial cells lead to release of chemokine CXCL8 (IL-8) and colony stimulatory factors (CSF), which further recruit and activate neutrophils thus increasing inflammation in the airway [151, 154, 157]. In addition, IL-17A also acts on endothelial cells and fibroblast to secrete the IL-1β and IL-6 for neutrophil recruitment [158]. Additionally, IL-17F act on fibroblasts and increase the expression of smooth muscle actin, thereby contributing to the remodeling of airways [153, 154]. Although severe asthma is caused by both Th2 and Th17, it is the Th17 mediated pathology which is mainly unresponsive to corticosteroid therapy [159, 160]. Recent reports suggest that EVs released by all these cell types in lungs are involved in airway inflammation and may be involved in allergic reactions through paracrine secretions [102, 161–174]. Pathogenic effect of EVs derived from *Staphylococcus aureus* that is present in house dust, was shown to stimulate Toll-like receptor-2 dependent Th1 and Th17 induced airway inflammation and augment the hypersensitivity response to the inhaled ovalbumin allergen [175].

Extracellular vesicles released from B-lymphocytes carry major histocompatibility complex (MHC) co-stimulatory molecules, antigenic peptides, [167] and HSP70 [176] which trigger inflammatory T cell responses. These EVs can also stimulate antigen-presenting cells to generate immune response by releasing Th2 inflammatory cytokines that contribute to AHR, critical to the development of asthma [167, 177]. Proteomic analysis of exosomes derived from LPS-stimulated and unstimulated equine neutrophils suggested presence of proteins known to play various roles in innate immunity, immune regulation, metabolism, and membrane trafficking [178]. These analyses also revealed the presence of chaperone proteins known to be associated with asthma remodeling [178]. Neutrophil-derived exosomes were reported to be internalized into pulmonary smooth cells and release bio-effector molecules that lead to their proliferation and promote airway remodeling and asthmatic progression [178].

Eosinophil-derived EVs have been shown to be released in high amounts in asthma patients that may be involved in airway inflammation [100] (Table 1**)**. These EVs carry various proinflammatory molecules such as interleukins, chemokines, chemotoxins like RANTES and eotaxin-1, prostaglandins, and platelet activating factors that are known to promote AHR and asthma pathogenesis [162, 179, 180]. Another study demonstrated that eosinophil-derived exosomes from the blood of asthma patients induced apoptosis in primary alveolar epithelial cells at 24 h and 48 h of exposure, via reduction in JAK/STAT signaling, and stimulating the release of inflammatory mediators like TNF and CCL26 [101]. However, on further exposure, these EVs promote epithelial proliferation by activating PI3/AKT signaling [101]. Furthermore, eosinophil-derived EVs induce proliferation of bronchial smooth muscle cells and increase VEGF-A and CCR3 expression in these cells through activation of ERK1/2 signaling, thus contributing to the fibrosis and remodeling observed in asthma [101].

Extracellular vesicles released from alveolar epithelial cells are reported to be higher in asthmatic mice as compared to control mice [164]. In animal models, lung epithelial cell-derived exosomes enhance the infiltration of macrophages and production of IL-13, leading to the development of asthma [164]. Another study demonstrated that human tracheobronchial epithelial cells change their protein and miRNA expression pattern after the uptake of exosomes from alveolar epithelial cells with asthma pathology, and may be responsible for higher mucin secretion and airway remodeling [181]. The EVs isolated from bronchial alveolar lavage fluid (BALF) have been widely studied for its role in asthma. The role of BALF EVs in the pathogenesis of asthma and allergic diseases was first reported by Admyre et al. [182]. In BALF from asthma patients, there is an increased production of EVs that directly correlates with increased HLA-DR expression responsible for immune activation of lung cells during the disease [102, 104] (Table 1**)**. These exosomes have also been demonstrated to carry high levels of functional proteins involved in leukotriene production [102]. Additionally, an increased level of BALF EVs among asthmatics is positively correlated with eosinophil and IgE levels in blood along with more CD54^+^ EVs for cell adhesion [104]. Further, when these BALF exosomes were exposed to bronchial epithelial cells, they induced production of IL-8 and leukotrienes, potent proinflammatory mediators in the recipient cells [102]. Another study showed that exosomes isolated from BALF of asthmatic patients were positive for tissue factor VIII, an important factor that plays role in coagulation and promoting angiogenesis [183].

Circulating EVs from BALF and plasma may also carry miRNA cargo that may be contributing to the pathogenesis of asthma and also could serve as biomarkers for the disease [184]. A study from Sweden characterized miRNA content of the exosomes isolated from the BALF of asthma patients [185]. These investigators found alteration in the levels of 18 miRNAs in asthma patients when compared with healthy controls, out of which 8 miRNAs (let-7a, miRNA-21, miRNA-658, miRNA-24, miRNA-26a, miRNA-99a, miRNA-200c, and miRNA-1268) showed significant alteration in expression [185] (Fig. 2). A strong correlation was observed between the expression profile of these altered miRNAs and FEV1 within the asthmatic patient group [185]. Let-7 family miRNAs have been shown to be influential in respiratory inflammation and AHR through regulation of IL-13 secretion. Using an established murine model of allergic airway inflammation, Kumar et al. showed significant reductions in let-7 miRNAs in allergic inflammation lungs compared to healthy controls. After uptake by let-7 miRNAs in the murine lung, significant reductions in IL-13 levels were observed in tissue, BALF, and serum. This correlated with a significant reduction in AHR in response to methacholine [186]. Therefore, let-7-miRNAs delivered via EVs may serve as a potential therapeutic strategy for AHR in asthma that warrants further research. MicroRNA-21 has been shown to be induced by IL-13, which is critically responsible for airway hyperreactivity, in the ovalbumin (OVA) murine model of asthma [187, 188]. MicroRNA-21 also contributes to polarization of helper T cells toward a Th2 phenotype, further supporting the important role of miR-21 in the hyperinflammation characteristic of asthma [187]. Given the influence of miR-21 in AHR and the pathogenesis of asthma, future exploration of the role of exosomal miR-21 as a biomarker for disease and FEV1 decline is warranted [187, 189, 190]. MicroRNA-140-3p is an important regulator of expression of chemokines and CD38, and appears to play an influential role in airway smooth muscle cell hyperplasia [191–193]. Circulating exosomes of patients with severe asthma exhibit significant upregulation in miR-140-3p compared to patients with mild-to-moderate asthma and healthy controls, suggesting that this miRNA may serve as an important prognostic biomarker. This study also demonstrated upregulation in miR-128, miR-196b-5p, and miR-486-5p in severe asthma patients, although the functional role of these miRNAs in the pathogenesis of asthma has not yet been elucidated [194].

Recent study signifies the role of functional mitochondria transfer via exosomes between different cell types in the disease pathogenesis [103]. Hough et al. reported the presence of mitochondria in the EVs from Bal fluid and from MHC class II cell surface receptor positive (HLA–DR^+^) myeloid-derived regulatory cells. The HLA–DR^+^ EVs isolated from asthmatic patients had increased number of mitochondria and more amount of mitochondrial DNA within its cargo. Additionally, uptake of these mitochondria carrying EVs by CD4^+^ T cells resulted in the generation of ROS and increased T cell proliferation.

### Lung cancer

Extracellular vesicles have been implicated in mediating intercellular communication responsible for lung carcinogenesis. Lung cancer is divided into two broad categories - small cell lung cancer (SCLC) and non–small cell lung cancer (NSCLC). The NSCLC is further divided to 3 subtypes: adenocarcinoma, squamous cell carcinoma, and large cell carcinoma [195]. The major reasons for development of lung cancer are smoking [196], genetic factors [197], toxic/carcinogen/environmental pollutants exposure (such as dust, asbestos), gender [198], and diet [199]. In addition, various types of bacterial and viral infections including HIV-1 and HPV can lead to the development of lung cancer [200, 201].

The EVs may contribute to various cellular functions, including epithelial-mesenchymal transition, angiogenesis, tumorigenesis and metastasis associated with lung cancer [202, 203]. Further, exosome secretion and release within the tumor microenvironment changes the levels of cytokines and growth factors such as TGF-β, IL-10, IL-6, MCP-1 by activation of proliferative signaling cascades like MAP kinases and NF-KB pathways and are therefore instrumental in promoting lung tumor progression and metastasis [204–206]. Exosomes released from metastatic small cell lung cancer cells carry increased amounts of TGF-β and IL-10 and are able to induce cancer cell proliferation and migration [205]. Tumor microenvironment derived exosomes also reprogram the metabolic machinery to provide metabolites like amino acids, lipids, and respiratory cycle intermediates that can be utilized by cancer cells for their growth and metabolism under nutrient-stress conditions [207]. Rab27a facilitates exosomes in carrying pro-inflammatory cytokines and matrix metalloproteinases (MMPs) responsible for tumor progression and cancer cell metastasis [208]. Microvesicles derived from activated platelets can also cause metastasis of lung carcinoma. Additionally, exosomes have been shown to carry enzymes which can synthesize their respective products in the recipient cells and modify the cellular phenotype [209]. The exosomes from the pleura exudates of lung cancer patients showed the presence of leukotriene synthesizing enzyme γ-glutamyl transpeptidase-1 which synthesizes the pro- tumorigenic LTD4 leukotriene from LTC4 produced by monocytes to support cell survival and migration of tumor cells [209]. Platelet-derived MVs contain integrin and matrix metalloproteinases that cause hyperproliferation and enhanced migration of lung cells, along with increased expression of angiogenic factors such as VEGF, MMP-9, and IL-8 [210]. These MVs also lead to increase in metastasis and angiogenesis in human syngeneic mice [210]. Platelet-derived MVs from lung cancer patients are also able to induce metastasis and promote tumor cell invasion by delivering miR-223 that targets tumor suppressor gene EPB41L3 (erythrocyte membrane protein band 4.1-like 3) [210]. Via mRNA and miRNA, cargo of exosomes derived from lung cancer cells can also stimulate the normal lung cells to undergo epithelial-to-mesenchymal transition [105] and induce migration and proliferation [206, 211]. Tumor cell-derived exosomes have been reported to contain hTERT mRNA responsible for increased telomerase activity within cells that causes uncontrolled cell growth and malignancy [212]. This hTERT mRNA gets transported via exosomes to non-cancer cells where it induces cell proliferation and delayed senescence, producing cancer-like characteristics [212]. Interestingly, HIV-derived transactivation response element (TAR) RNA has been known to activate proto-oncogenes and TLR3 inducible genes, promote tumor growth and cancer cell progression [213]. It was observed that exosomes from HIV-infected T cells carrying HIV TAR RNA enter lung cancer cells through epidermal growth factor receptor (EGFR), stimulating cell proliferation and migration through activation of the ERK1/2 signaling pathway [213].

Exosomes derived from neoplastic transformed umbilical vein endothelial cells can transfer miR-21 to normal cells and lead to increased angiogenesis and malignant transformation through increased activation of STAT3 signaling and VEGF levels [214]. miR21-5p also inhibits PTEN in lung cancer cells to activate AKT signaling pathway and promote tumor cell growth, cell proliferation, and epithelial-mesenchymal transition [215]. Exosomal miR-21 and miR-155 have been reported to be more highly expressed in recurring lung cancer tumors than primary tumors, suggesting that specific miRNA signatures in exosomes derived from serum of lung cancer patients can serve as biomarkers for diagnosis of disease [215]. Hypoxic lung cancer cell-derived exosomes carry high levels of miR-23 that targets prolyl hydroxylase and zona occludens-1 in endothelial cells [216]. Disruption of tight junction proteins induces vascular permeability, angiogenesis and trans-endothelial migration of cancer cells [216]. Tumor-derived exosomes induce differentiation of fibroblasts to tumor-promoting stromal fibroblasts by activation of TGF-β signaling [217]. However, another study found that lung adenocarcinoma cell-derived EVs deliver miR-142-3p to endothelial cells to promote angiogenesis, cell proliferation and differentiate fibroblasts to cancer-associated phenotype, independent of TGF-β signaling [218] (Fig. 2). Notably, EVs derived from the fluid of pleural effusions in lung cancer patients also exhibit alterations in miRNA cargo [218]. One study reported the presence of miR205-5p and miR-200b in high amounts in lung cancer patients [219]. Another study demonstrated that patients with malignant lung adenocarcinoma have high circulating levels of miR-182 and miR-210 in pleural effusion, which is noticeably absent in benign lung cancers suggesting an involvement in lung cancer progression [220]. Serum-derived EVs from high-grade malignant lung cancer patients have high expression of miR-96, a tumor promoter [221]. MiR-96 mainly acts by targeting LIM-domain only protein 7 (LMO7) protein that helps in maintaining alveolar architecture, actin cytoskeleton and functions as tumor suppressor in lung cancer [221].

In addition to miRNA cargo, long non-coding (lnc) RNAs are also carried by EVs which may contribute to lung cancer progression and tumorigenesis. The lncRNA MALAT-1 (metastasis associated lung adenocarcinoma transcript-1) is found to be highly up-regulated in the exosomes derived from serum of lung cancer patients and expression is also correlated with the metastatic stage [106] (Table 1**)**. MALAT-1 was also able to prevent lung cancer cell apoptosis, alternatively leading to proliferation, cell migration and invasion [106]. LncRNA H19 is highly expressed in geftinib- resistant lung cancer cells and gets packaged into their exosomes [222]. Geftinib is a tyrosine kinase inhibitor used as a therapy for highly malignant non-small cell lung cancer (NSCLC) and geftinib-resistance is one of the major obstacles in its treatment [222]. In this study, it was shown that lncRNA H19 is delivered to non-resistant cells via exosomes and able to confer geftinib-resistance [222].

### Acute lung injury (ALI), acute respiratory distress syndrome (ARDS), and pulmonary Sepsis

Acute lung injury (ALI) and acute respiratory distress syndrome (ARDS) is characterized by inflammation and disruption of the endothelial and epithelial barriers of the lung, leading to acute respiratory failure and a very high mortality rate [223]. The most common cause of ALI and ARDS is sepsis secondary to pulmonary infection [224]. Lately, there has been a profound increase in reports demonstrating the influential role of EVs in sepsis-induced and non-sepsis-induced ALI and ARDS. Leukocyte-derived MPs circulating in BALF and blood of ARDS patients appear to be associated with patient survival [107] (Table 1**)**. Additionally, endothelial cell-derived MPs have also shown promise as biomarkers and mediators of ALI and ventilator-induced lung injury (VILI) [107]. In vitro and in vivo studies have shown increased release of endothelial-derived microparticles (EMPs) representative of cellular dysfunction, after exposure to mechanical stress and endotoxin characteristic of Gram-negative bacterial infection [225]. Mechanical stress-derived EMPs were also able to develop inflammation and injury in lungs when injected in healthy mice [225]. These EMPs have been reported to be released by endothelial cells during ALI due to defects in cytoskeleton. Their increased levels were induced by external or internal stimuli such as LPS which corresponded to the decline in the surface area of plasma membrane and cell volume, enlarging the intercellular gaps and junctions [226]. Another study showed that IFN-α induced pulmonary injury resulted in an increase in the number of circulating EMPs in the blood, leading to cytoskeleton rearrangement and endothelial cell apoptosis [227]. This was prevented by inhibition of Rho-kinase activity and targeting EMPs may be useful as well [227]. In an ARDS rat model, Li et al. found no difference in total blood MPs versus controls; however, the concentration of leukocyte- and endothelium- derived MPs was higher in ARDS, further demonstrating their influential role in the pathogenesis of ARDS [228].

Encapsulated caspase-1 in monocyte-secreted MPs is able to induce apoptosis of human pulmonary microvascular endothelial cell (HPMVEC) in ALI/ARDS [108, 229]. These findings are in agreement with other reports on traumatic brain injury (TBI) induced ALI/ARDS, where higher levels of ASC (apoptosis-associated speck-like protein containing a caspase-recruiting domain) in the serum EVs led to the pyropotosis of endothelial cells via activation of inflammasomes [109] [230]. EMPs have also been shown to initiate a cascade of pulmonary and systemic proinflammatory molecules leading to the development of lung injury [231]. EVs released by lung epithelial cells in the hyperoxia-induced ALI (HALI) animal model were found to activate alveolar macrophages to induce proinflammatory response in lung tissue upon delivery of caspase-3 [232]. Another study showed the role of MVs from BALF and lung epithelial cell-culture media in HALI with significant alterations in the levels of miR-320a and miR-221 responsible for proinflammatory response via macrophage activation [233] **(**Fig. 2**)**. Studies involving LPS-induced ALI showed the rapid production of pro-inflammatory MVs and exosomes by lung macrophages, leading to lung injury [234, 235]. Also, endothelial and leukocyte-derived MPs in blood from LPS-treated rats were shown to induce a proinflammatory response and ARDS in healthy rats [236, 237].

In influenza virus-induced ALI, EVs have been shown to suppress antiviral factor and promote replication of influenza virus through a miRNA-mediated mechanism in lung epithelial cells [238]. In acid-induced ALI, the elevated levels of miR-17 and 221 in lung epithelium-derived MVs were found to be involved in the activation and recruitment of macrophages [239]. Both sterile stimuli (oxidative stress or acid aspiration) and infection (LPS/Gram-negative bacteria) lead to increase in BALF EVs, however, the source for these EVs differed [240]. BALF EVs from sterile stimuli was mainly from alveolar type-І epithelial cells, whereas infection-induced BALF EVs were from alveolar macrophages (AMs). Nonetheless, both kinds of EVs generated same kind of functional response by promoting macrophage recruitment and generation of inflammatory cascade in the lungs [240].

In addition to the pathways previously reviewed, aberrations in coagulation and fibrinolysis also play pivotal roles in inducing inflammatory responses in ALI and ARDS. Tissue factor (TF), an initiator of the coagulation cascade, is detected in high levels in patients’ lungs and can cause deposition of fibrin in airspace [110]. MPs containing TF are released by alveolar epithelium in response to pro-inflammatory stimulus in ALI/ARDS lungs, therefore contributing to coagulation [110] (Table 1**)**.

Most studies analyzing EVs in patients with sepsis have included those with undifferentiated sepsis due to multiple potential sources, making generalizability of findings specific to pulmonary sepsis difficult. There is some evidence to indicate differences in EV cargo exist based on source of underlying sepsis. Researchers have shown that plasma-derived MVs from patients with sepsis due to community-acquired pneumonia (CAP) have higher expression of alpha-2-macroglobuin compared to patients with sepsis due to fecal peritonitis [111]. Furthermore, they demonstrated an association between higher plasma alpha-2-macroglobulin positive MVs and survival in patients with CAP [111]. This was consistent with previous findings from the same group, in which granulocyte-derived EVs carrying alpha-2-macroglobulin were protective in patients with sepsis due to CAP [112] (Table 1**)**. These results are noteworthy in consideration of the fact that the cecal ligation and puncture (CLP) model is commonly used as a mouse model of sepsis [241]. Results generated from CLP models may not be generalizability to non-peritonitis subsets of sepsis, including sepsis secondary to lung infections. Further research on the dynamics of circulating EVs in patients with sepsis and evaluation of differences based on source of infection and causative pathogen are warranted.

## Therapeutic potential of EVs in lung diseases

Though EVs are implicated in the pathogenesis of the pulmonary complications, as we have reviewed; EVs also may serve in a role as potential therapeutic agents (Table 2) [161, 206, 261, 262]. Mesenchymal stem cells (MSC) and the products released from these cells such as MSC-derived extracellular vesicles (MSC-EVs) are being explored for their protective capabilities against lung diseases [262–264].

**Table 2:** Therapeutic role of extracellular vesicles in various lung complications

Disease	EV source	EV type	Cargo molecule (s)	Major effects /role	Ref.
COPD	Adipose-derived stem cells	Artificial nano-vesicles	FGF2	Increase in epithelial cell proliferation, inhibition of emphysema and regeneration of damaged lung of mice	[22]
PH	Mesenchymal stromal cell	Exosomes	–	Reduction in vascular remodeling and hypoxic PH, inhibition of pro-proliferative STAT3 signaling in pulmonary arterial endothelial cells	[242]
	Mesenchymal Stem Cells	MVs	–	Reduction in mean pulmonary arterial pressures, right ventricle hypertrophy in monocrotaline-PH rat model	[243]
	Mesenchymal stromal cell	Exosomes	–	Promoted mitochondrial function and TCA cycle in pulmonary artery smooth muscle cells	[244]
	Mesenchymal Stem Cells	Exosomes	miRs-34a, −122, −124, and − 127	Reversed PH in monocrotaline mice model	[126]
	Mesenchymal Stem Cells	EVs	–	Reversal of bone marrow endothelial progenitor cells (EPCs) mediated PAH	[129]
	Endothelial cells	MPs	Endoglin	Improved survival and proliferation of pulmonary endothelial cells	[245]
Asthma	Mesenchymal Stem Cells	Exosomes	–	Promoted proliferation and immune-suppression capacity of T regulatory cells	[246]
	Adipose derived Mesenchymal Stem Cells	EVs	–	Reduced airway remodeling and eosinophil counts in lung tissue and BALF of ovalbumin mice.	[247]
	Mesenchymal stromal cells	EVs	–	Abrogated inflammatory response by increasing IL-10 and reducing Th2 and Th17 associated cytokines in the mice model of asthma	[248]
	Bone marrow derived mast cells	Exosomes	IgE receptors (FcξR1)	Reduced IgE levels and mast cell activation in allergic asthma mouse model	[172]
	Human bone marrow derived mesenchymal stem cells	EVs	–	Prevent development of airway hyper responsiveness and pulmonary inflammation in response to allergen	[248]
	*Pseudomonas aeruginosa*	Exosomes	–	Prevention of allergic reactions by increasingTreg and decreasing the Th2 response.	[249]
	Human mesenchymal stromal cells	Small EVs	miR-146a-5p	Reduction in the infiltration of inflammatory cells, Th2 cytokines and airway hyperresponsiveness	[250]
Lung Cancer	Dendritic cell derived exosomes (DEX)	Exosomes	MAGE tumor antigen	Modest stabilization of NSCLC patients in response to DEX immunotherapy	[251]
ALI/ARDS	Mesenchymal Stem Cells	MVs	Keratinocyte Growth Factor mRNA	Reduction in pulmonary edema and influx of inflammatory cells in BAL of *E. coli* endotoxin –induced ALI mice;	[252]
	Mesenchymal Stem Cells	Exosomes/MVs	Mitochondria/ miRNA	MSC-MVs transfer depolarized mitochondria to macrophages and increase macrophage bioenergetics; MSC-exosomes modulate TLR signaling and cytokine release in macrophages	[253]
	Mesenchymal stromal cells	EVs	Mitochondria	Reduced inflammation and lung injury; enhanced oxidative phosphorylation in macrophages	[254]
	Mesenchymal Stem Cells	EVs	Runx1 p66 and p52	Enhanced junctional integrity of injured endothelial cells and decreased lung pathology	[255]
	Mesenchymal Stem Cells	EVs	–	Modulated cytoskeletal signaling in endothelial cells and attenuated lung vascular permeability	[256]
	Mesenchymal Stem Cells	MVs	–	Increased alveolar fluid clearance and reduced protein permeability and inflammation; increased antimicrobial in ex-vivo perfused human lung model of bacterial pneumonia	[257]
	Umbilical cord mesenchymal stromal cells	Exosomes	Angiopoietin 1 and hepatocyte growth factor	Restoring alveolar fluid clearance and protein permeability of influenza virus infected alveolar epithelial cells	[258]
	Endothelial progenitor cells	Exosomes	miR-126	Enhanced proliferation, migration of endothelial cells by promoting RAF/ERK signaling, ameliorated LPS-induced lung injury	[259]
	Inducible pluripotent stem cells	Exosomes	siRNAs against ICAM-1	Successfully delivered siRNA into HMVECS and inhibited expression of ICAM-1 and neutrophil adhesion	[260]

### COPD/asthma

When administered to activated alveolar macrophages, MSC-EVs lead to reduction in the release of proinflammatory molecules, decrease the infiltration of neutrophils and lymphocytes in the BALF and airways, and contribute to reducing the collagen content in the lung parenchyma [253, 254, 263]. Overall, this leads to a protective effect against lung disease like COPD and ALI [253, 254, 263]. Moreover, MSC-EVs are able to transfer mitochondria to alveolar epithelial cells and macrophages to overcome oxidative stress characteristic of lung diseases like COPD and ALI [253, 254].

MSC-derived EVs have been shown to exert immunomodulatory effects on PBMCs, including release of IL-10 and TGF-β, stimulating proliferation of T regulatory cells (Tregs) and leading to immune suppression in asthmatic patients [246]. Adipose tissue derived-mesenchymal stem cells EVs (AD-MSC) reduce eosinophilic infiltration into the lung tissue and parenchymal collagen content in mouse models of asthma [247]. MSC-derived EVs have also been found to abrogate inflammatory response by increasing anti-inflammatory IL-10 and reducing Th2 and Th17 associated cytokines [248]. Kim et al. prepared AD-MSC-derived artificial nanovesicles that expressed similar AD-MSC surface markers and growth factors such as FGF2, important in lung regeneration [22]. These nanovesicles were able to induce epithelial cell proliferation via FGF2-dependent pathway and inhibit emphysema in mice model [22].Mast-cell derived exosomes exhibit FcξR1 receptors that trap free IgE and limit the effects of mast-cell activation during asthma [172]. Another study showed protective effects of EVs from human bone marrow derived mesenchymal stem cells in preventing the development of AHR, pulmonary inflammation and Th2 and Th17 antigen dependent activity in the allergic response to Aspergillus hyphal extract in immunocompetent mice [248]. The therapeutic utility of human mesenchymal stromal cells derived small EVs in reducing allergic airway asthma was also demonstrated by their ability to lower the levels of group 2 innate lymphoid cells (ILC2s), reduce AHR and infiltration of inflammatory cells with decrease in the levels of Th2 cytokines and mucus production in lungs [250]. Furthermore, the inhibitory effect of exosomes isolated from *Pseudomonas aeruginosa* on the development of AHR along with reduced inflammation in the perivascular and peribronchial spaces in allergic asthma model has also been reported. This inhibitory effect was attributed to the increased Treg and attenuated Th2 responses on treatment with *P· aeruginosa* derived exosomes [249].

### PH

Mesenchymal stromal cell-derived exosomes were able to cause reduction in right ventricle systolic pressure and vascular remodeling in a mouse model of hypoxia-PAH by inhibiting induction of proinflammatory and pro-proliferative mediators and macrophage influx by suppressing the hypoxic activation of STAT3 and miR-17 super family [242]. In addition, these exosomes increased the expression of anti-proliferative miR-204 in lungs of PAH mice [242]. Similarly, MSC-derived MVs were able to ameliorate PAH in the MCT-PAH rat model via reduction in right ventricular hypertrophy, mean right ventricular and mean pulmonary arterial pressures, and decreased pulmonary arteriole remodeling [243]. Mesenchymal stromal cells were able to promote the mitochondrial function and led to increased expression of pyruvate dehydrogenase (PDH) and glutamate dehydrogenase 1 (GLUD1), thus promoting the citric acid cycle in pulmonary smooth muscle cells [265]. When injected to MCT-PAH mice, MSC-exosomes reduced and reverted PAH complications [126]. These exosomes were rich in anti-inflammatory, anti-apoptotic, and anti-proliferative miRNAs such as miR –34a, − 122, − 124, and − 127 [126]. MSC-derived EVs have also shown to reverse the effect of endothelial progenitor cells from PAH mice in developing PAH in healthy mice [129]. Increased levels of circulating endoglin+ endothelial MPs in the plasma of chronic thromboembolic pulmonary hypertension (CTEPH) patients improved the survival and proliferation of recipient cultured primary human pulmonary endothelial cells, thus demonstrating a therapeutic role of these particles [245]. Apart from MSCs, delivery of miR-195 to SMCs via endothelial-derived exosomes was reported to prevent SMC proliferation and migration by inhibiting the expression of serotonin transporters and thus may play a protective role in PAH [266].

### Lung Cancer

Exosomes isolated from dendritic cells called dexosomes have successfully covered the journey from the animal models in laboratory to patient bedside as an immune system amplifying tool to treat NSCLC. The isolation of dexosomes from patient blood and then loading them with antigens, MHC I and II molecules and re-administration to patients has proved to be a successful way to stimulate naive dendritic cells (DCs) in patients and boost up both innate and adaptive immune system in fighting lung and other cancers [251, 267, 268]. In addition, loading them with B and T cell epitopes enhances their immunogenicity [269]. Circulating tumor EVs can also potentially act as carriers of anti-tumor drugs, small interfering RNAs, and molecules like anti-programmed cell death receptor 1 (PD-1) and anti-programmed cell death ligand 1 (PD-L1) to prevent lung cancer progression [270].

### ALI/ARDS/pulmonary Sepsis

MSC-derived EVs from ARDS patients showed the presence of transforming growth factor-beta receptor I (TβRI)/Alk5 and the Runx1 p66 and p52 transcription factor that are crucial in protecting ARDS [255]. Importantly, higher Runx1p66/p52 ratio provided a survival advantage [255]. Runx1p66 from bone marrow-derived MSC-EVs induces proliferation of LPS-treated ECs and help to improve pathology of lung in LPS induced ALI mice [255]. Bone marrow-derived mesenchymal stem cell EVs induced cytoskeletal RhoA GTPase activity, leading to a significant decrease in hemorrhagic shock-induced lung vascular permeability in the hemorrhagic-shock mice model of ARDS [256]. Human MSC-derived EVs were also able to ameliorate ALI secondary to *E. coli* bacterial pneumonia by reducing lung protein permeability and pulmonary edema and improving alveolar fluid clearance, leading to a reduction in both bacterial load and median pulmonary artery pressure [257]. In addition, umbilical cord mesenchymal stromal cell-derived exosomes were found to suppress influenza virus-induced ALI by improving the clearance of alveolar fluid and protein permeability of A(H5N1)-infected human alveolar epithelial cells [258]. Another report demonstrated transfer of functional mitochondria by MSC derived EVs to the recipient macrophages [254]. These CD44 expressing MSC-derived EVs acted as a successful therapy in the LPS model of lung injury by suppressing cytokine production, inducing anti-inflammatory response, and reducing lung pathology through enhanced oxidative phosphorylation in macrophages [254]. Exosomes released from endothelial progenitor cells (EPCs) have also been reported to improve the injury in LPS-induced ALI rat model by regulating integrity, migration, and proliferation of ECs. EPC exosomes mediated transfer of miR-126 to ECs targeting the expression of SPRED1 and enhancing RAF/ERK signaling pathways were primarily responsible for restoring lung health in this model [259]. Human-induced pluripotent stem cell-derived exosomes have been used as delivery systems for siRNAs targeting ICAM-1 in human primary pulmonary microvascular endothelial cells (HMVECs), leading to obstruction of ICAM-1 protein expression and inhibition of neutrophils-endothelium adhesion induced by LPS, the primary features of ALI [260].

## Summary

In conclusion, EVs are emerging as vital components of multiple pulmonary pathologies. It is highly apparent that EVs represent a heterogeneous population that differs substantially in composition and its cargo. Most of the studies included in this review investigated the role of miRNA cargo in the lung pathogenesis related to the respective diseases (Fig. 2), however the role of other small non-coding RNAs needs equal attention and should be the focus of future investigations. Additionally, various other EV content such as proteins, cytokines, enzymes are equally important to be explored further. The study of EVs is a rapidly evolving field and there remains a lack of uniformity in methods used to isolate and categorize EVs. Inconsistencies across studies may substantially influence results, which limit the generalizability of any individual study. Standardization of these methods and validation of findings in multiple prospective longitudinal cohorts is essential for the field to continue to move forward and positively impact patient care. Furthermore, pulmonary diseases are physiologically complex and occur on a spectrum of severity, which adds an additional layer of difficulty to generalize the findings from these studies. For example, there are many subtypes of lung cancer that differ in underlying molecular pathology, treatment, and prognosis [271]. This heterogeneity is true for nearly all pulmonary diseases and future investigations evaluating EVs as biomarkers and therapeutics should clearly account for these differences. Despite these limitations, further identification of EVs and corresponding cargo has the potential to aid in the discovery of clinically useful biomarkers and the development of novel therapeutics for lung pathologies. This promising area of research should provide some hope to the millions of patients suffering from lung diseases which are currently incurable by today’s standard of care.

## Data Availability

All publications discussed in the manuscript are available from the corresponding author on request.

## Abbreviations

EVs: Extracellular Vesicles
COPD: Chronic Obstructive Pulmonary Disease
ALI: Acute Lung Injury
ARDS: Acute Respiratory Distress Syndrome
MVB: Multivesicular Bodies
ESCRT: Endosomal Sorting Complexes Required for Transport
MVs: Microvesicles
MPs: Microparticles
lncRNA: Long non-coding RNA
FEV: Forced Expiratory Volume
TF: Tissue Factor
BALF: Bronchoalveolar Lavage Fluid
EMPs: Endothelial-derived Microparticles
PH: Pulmonary Hypertension
PAH: Pulmonary Arterial Hypertension
IPAH: Idiopathic Pulmonary Arterial Hypertension
HPASMCs: Human Pulmonary Arterial Smooth Muscle Cells
MCT-PAH: Monocrotaline-induced Pulmonary Arterial Hypertension
AHR: Airway Hyper Responsiveness
ECs: Endothelial Cells
PMVECs: Pulmonary Microvascular Endothelial Cells
SMCs: Smooth Muscle Cells
NSCLC: Non–Small Cell Lung Cancer
MMPs: Matrix Metalloproteinases
MSC: Mesenchymal stem cells
EPCs: Endothelial Progenitor Cells
